# NKAIN1 expression relates to the immune evasion and prognosis of gastric cancer

**DOI:** 10.1038/s41598-025-30676-0

**Published:** 2025-12-06

**Authors:** Lindong Zhou, Yinfei Cheng, Peng Liu, Xiaoxia Jin, Qiang Xu, Xiaoxiang Li

**Affiliations:** 1 Department of Emergency, Rudong County People’s Hospital & Affiliated Rudong Hospital of Xinglin College, Nantong University, Jiangsu, 226400 China; 2 Department of Gastroenterology, Rudong County People’s Hospital & Affiliated Rudong Hospital of Xinglin College, Nantong University, Jiangsu, 226400 China; 3 Department of Oncology, Rudong County People’s Hospital & Affiliated Rudong Hospital of Xinglin College, Nantong University, Jiangsu, 226400 China; 4 Department of Pathology, Affiliated Tumor Hospital of Nantong University, Jiangsu, 226361 China

**Keywords:** NKAIN1, Gastric cancer, Tumor immune microenvironment, CTLA-4, Prognosis, Cancer, Computational biology and bioinformatics, Immunology

## Abstract

Patients with gastric cancer often exhibit poor responses to immunotherapy and experience unfavorable outcomes. This study aims to investigate the relationship between NKAIN1 levels in the tumor microenvironment and gastric carcinoma patients characteristics. Gene expression analysis, multiplex immunohistochemistry using tissue microarrays, and Quantitative Pathology Imaging System were conducted for determining the expression of NKAIN1 in gastric carcinoma tissues and association with patient outcomes. Additionally, we used the Spearman correlation method to assess the relationship between NKAIN1 protein expression in the tumor immune microenvironment and immune checkpoint markers of gastric carcinoma. Lastly, we evaluated the predictive significance of NKAIN1 presence using Cox regression models. The study findings indicated a significant increase in NKAIN1 expression in gastric carcinoma tissues compared to non-tumor stomach tissues. Moreover, a clear association between NKAIN1 expression and CTLA-4, as well as PD-L1, was identified. Importantly, the increased NKAIN1 protein expression was identified as an independent prognostic factor for adverse clinical outcomes in gastric cancer. In conclusion, this study supports NKAIN1 as a prognostic marker and promising target for immune therapy in stomach carcinoma.

**Supplementary Information:**

The online version contains supplementary material available at 10.1038/s41598-025-30676-0.

## Introduction

Gastric cancer is ranked fifth as the most prevalent malignancy worldwide and fourth as the leading cause of cancer-related mortality^1,2^. Its incidence has been observed to be notably high in East Asia and Eastern Europe^3^. Despite early diagnosis of gastric adenocarcinoma in Western Europe, the cure rate remains unsatisfactory, posing a significant burden on patients^4^. Current treatment modalities, including traditional surgery, chemotherapy, and emerging immunotherapies, have not substantially improved the prognosis for patients with stomach adenocarcinoma^5,6^. Therefore, understanding the composition of various cells in the tumor immune microenvironment (TIME) of gastric adenocarcinoma is crucial^5,7,8^.

While The Cancer Genome Atlas (TCGA) project has offered a substantial genomic dataset for gastric cancer, the analysis of the correlation between characteristics of the TIME and immune checkpoints holds promise for clinical research^9,10^. As molecular biology research on gastric cancer progresses, immunotherapy targeting the tumor microenvironment (TME) has garnered attention^11,12^. However, there remains an urgent need to leverage existing mRNA data to investigate immune-related prognostic markers or potential immunotherapy targets in gastric cancer^7,13^.

Our analysis of TCGA online data revealed a correlation between high expression of the Na+/K + transporting ATPase interacting 1 gene (NKAIN1) in gastric cancer tissues and patients’ poor prognosis. The NKAIN gene family, comprising NKAIN1, 2, 3, and 4, was initially identified as expressed in the central nervous system (CNS)^14^. As an evolutionarily conserved transmembrane protein, NKAIN1 interacts with the subunit of Na, K-ATPase β1 and is localized to neurons^14^. To date, limited research has been conducted on the NKAIN1 protein and its association with tumors, particularly in the context of clinical gastric cancer.

Given the complexity of biological samples, messenger RNA (mRNA) expression may not definitively predict high or low protein levels^15,16^. To elucidate the association between NKAIN1 protein expression in gastric cancer and clinical features, we conducted multiplex immunohistochemistry (mIHC) with gastric cancer tissue microarray (TMA). Our focus was on NKAIN1 protein levels within the TIME and its correlation with immune checkpoints. Overall, the study findings identify NKAIN1 as a prognostic indicator and a promising candidate for immunotherapy in gastric cancer.

## Materials and methods

### Bioinformatics analysis

TCGA database and Genotype-Tissue Expression RNAseq data for gastric adenocarcinoma (comprising 414 tumor samples and 210 benign samples) retrieved to determine the differential expression of NKAIN1 mRNA in gastric cancerous tissues compared to non-tumorous tissues and to evaluate the impact on patient prognosis. Our method was derived from Xiantao Academic (www.xiantaozi.com) and previous literature^17^.

### Clinical samples and data information

The TMA utilized in this study comprised 305 tumor tissues and 66 matched nonmalignant stomach tissues obtained from surgical margins. These samples were collected from the Affiliated Tumor Hospital of Nantong University between 2011 and 2017. Each core on the TMA represented a sample with a 2 mm diameter, as described in previous literature^18^. Clinicopathological and prognosis data of the samples, including age, gender, differentiation grade, HER-2 expression, and tumor stage, were extracted from the patients’ hospital records. Importantly, no patients included underwent immunotherapy, radiation therapy, or chemotherapy prior to surgery. Overall survival (OS) was delineated as the duration from the initial biopsy-confirmed diagnosis to the occurrence of death. Individuals who remained alive at the last follow-up date were censored from the analysis. Informed consent was obtained from all enrolled subjects in this research. Approval for the study protocol was obtained from the Human Research Ethics Committee of the local Hospital (ID: 2024-031). All methods and procedures followed the Declaration of Helsinki and local laws and regulations.

### mIHC staining with analysis

mIHC staining was conducted utilizing Opal 7-Color Manual IHC Kit (NEL810001KT; Akoya Biosciences, USA). Slices of the gastric cancer TMA were heated in a microwave with antigen retrieval and blocking buffers. Following incubation with the primary antibody, sections were treated with the Opal™ polymer HRP Ms + Rb (ARH1001EA; Akoya Biosciences, USA). Nuclei were stained with DAPI (F6057, Sigma, USA), following established protocols^18,19^.

The primary antibodies used in the study included anti-cytokeratin (CK) antibody (1:1000, orb69073, Biorbyt, UK), anti-NKAIN1 antibody (1:900, 105646, Abcam, UK), anti-CD3 antibody (1:200, 85061 S, CST, USA), anti-CD4 antibody (1:200, ab133616, Abcam, UK), anti-CD8 antibody (1:500, 85336 S, CST, USA), anti-CD20 antibody (1:100, ab78237, Abcam, UK), anti-CD66b antibody (1:500, arg66287, Airgobio, China), anti-CD68 antibody (1:1200, 76437 S, CST, USA), anti-CD86 antibody (1:1500, orb388891, Biorbyt, UK), anti-CD163 antibody (1:100, 93498 S, CST, USA), anti-Lamp3 antibody (1:500, ab111090, Abcam, UK), anti-PD-1 antibody (1:500, 86163, CST, USA), anti-PD-L1 antibody (1:100, 13684 T, CST, USA), and anti-CTLA-4 antibody (1:500, orb527271, Biorbyt, UK).

The fluorescence-stained TMA slides were scanned using the Quantitative Pathology Imaging System (Vectra 3.0, PerkinElmer, USA). The fluorescence signals corresponding to different proteins (NKAIN1 and immune markers) in each tissue core were recorded, scored, and quantified as a percentage value (positively stained cells/number of CK or DAPI staining, multiplied by 100%) using the matching software (inForm 2.6, PerkinElmer, USA).

### Statistical analysis

The Wilcoxon rank sum test was used for comparing NKAIN1 mRNA expression in gastric cancerous and nonmalignant stomach tissue. Pearson’s test was then conducted for examining the assocation of NKAIN1 protein expression with immune markers. To determine the cutoff point for NKAIN1 protein expression based on 5-year OS, we utilized X-tile (Yale University, USA)^20,21^. The chi-square test was used for determining the relationship of NKAIN1 protein levels with clinicopathologic characteristics. Prognostic factors were evaluated using Kaplan–Meier (K-M) with log-rank tests and Cox proportional-hazards models. All data analyses were performed using SPSS v20.0 (IBM Corporation, Armonk, USA). *P* < 0.05 and *R* > 0.2 were used for considering statistical significance.

## Results

### NKAIN1 mRNA in gastric carcinoma tissue

**Fig. 1:**
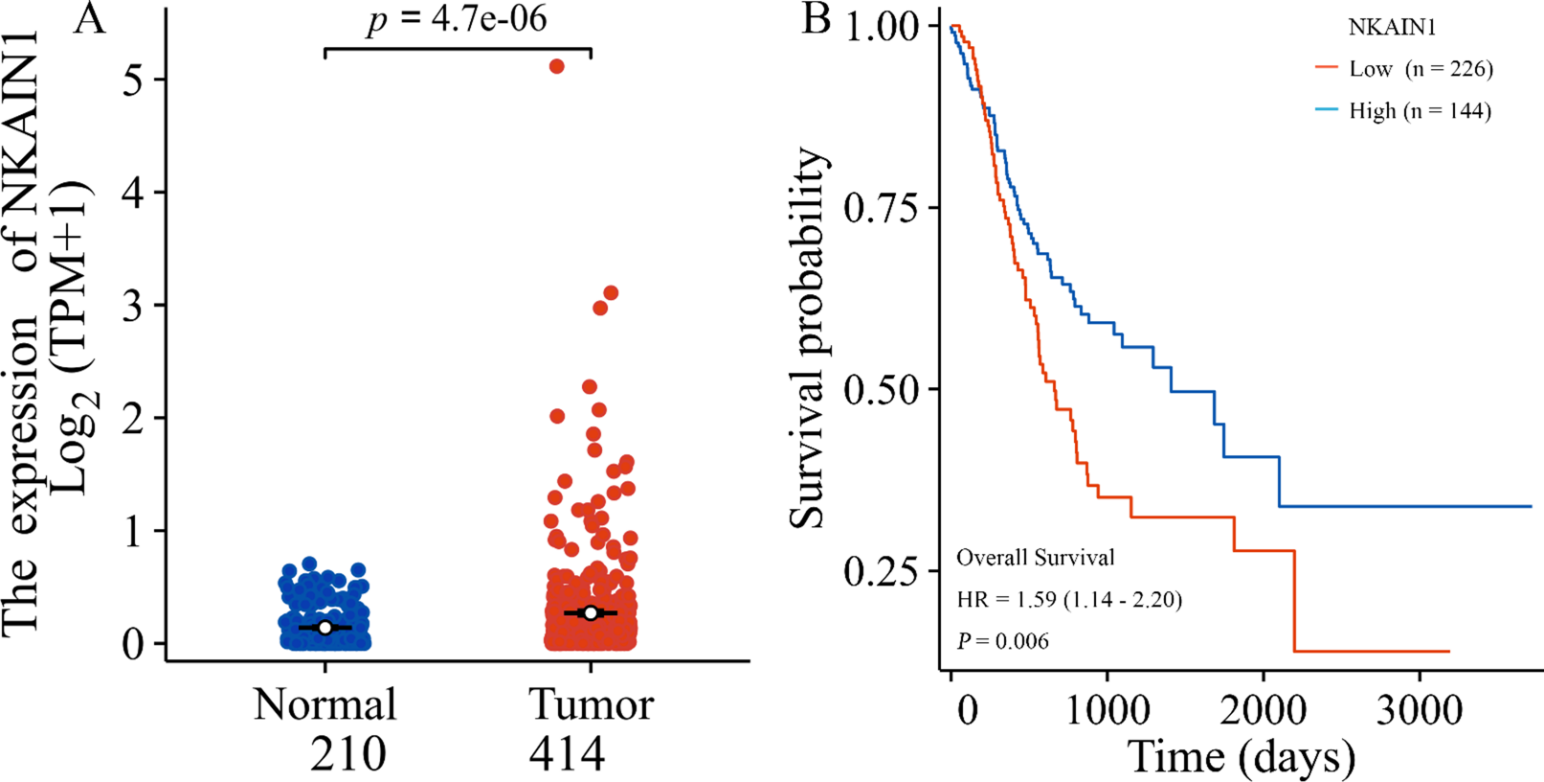
NKAIN1 mRNA expression in gastric carcinoma tissue from TCGA. (**A**) Comparison of NKAIN1 mRNA expression between stomach cancerous tissue (light maroon) and non-tumorous gastric tissue (bright green). (**B**) Kaplan-Meier survival curve analysis of gastric cancer patients stratified by NKAIN1 mRNA expression levels. High NKAIN1 mRNA expression (red) vs. low NKAIN1 mRNA expression (green).

The first objective of this study was to assess a potential actionable biomarker for gastric cancer. We hypothesized that the gene mRNA levels would be elevated in stomach cancerous tissues compared to non-tumorous gastric tissues (*P* < 0.001) and that high expression would correlate with an unfavorable prognosis (HR = 0.59, *P* = 0.006), as shown in Fig. 1.

### The NKAIN1 protein expression in the TME of stomach cancer tissues

**Fig. 2:**
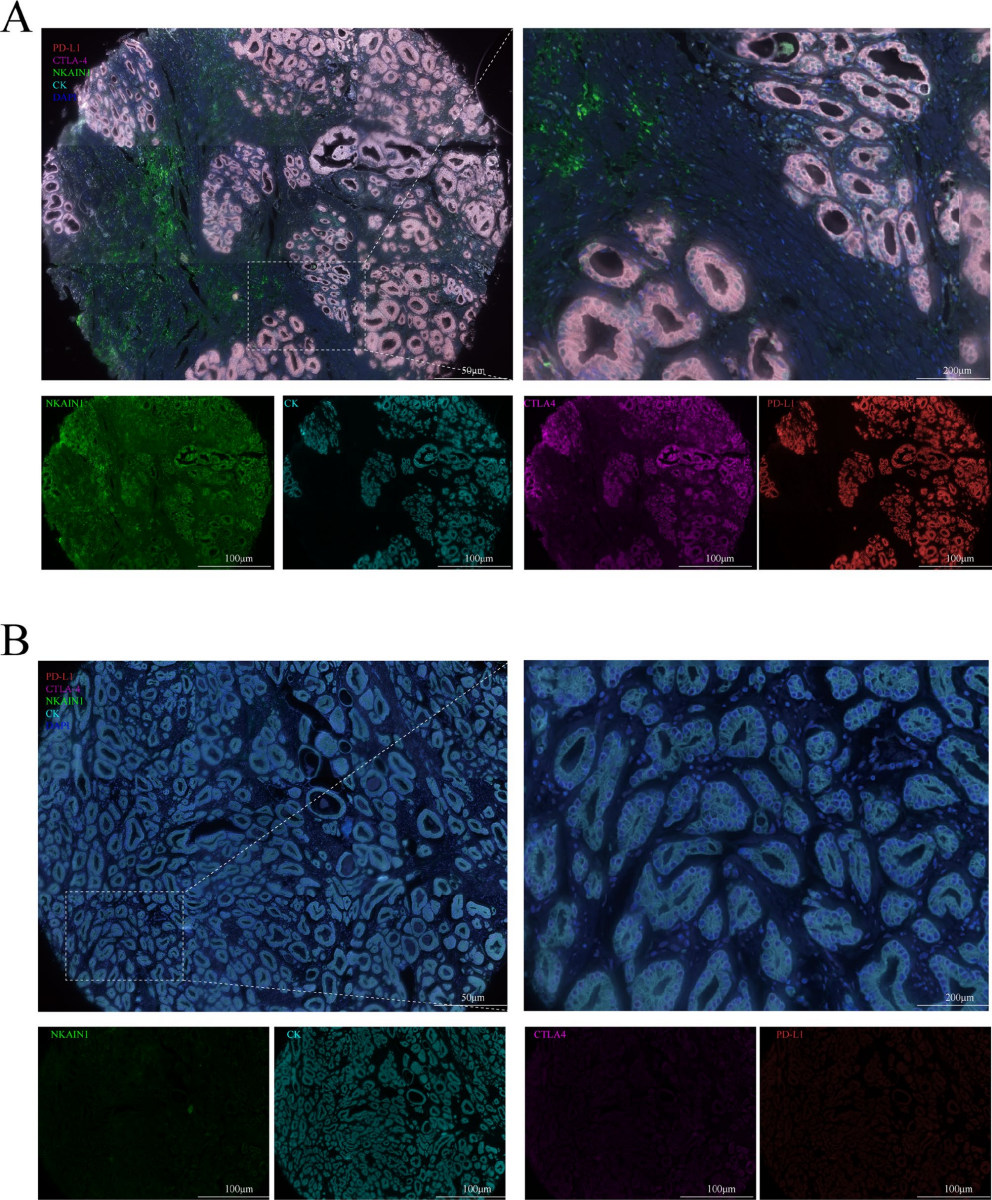
Representative multiplex immunohistochemistry images in gastric cancer tissue microarray (TMA). (**A**) High protein expression of NKAIN1, CTLA-4, and PD-L1 in gastric cancer tissues. (**B**) Low protein expression of NKAIN1 CTLA-4, and PD-L1 in nonmalignant stomach tissues. NKAIN1 (green), CTLA-4 (magenta), PD-L1 (red), Cytokeratin staining (aqua green) and nucleus staining with DAPI (blue).

**Fig. 3:**
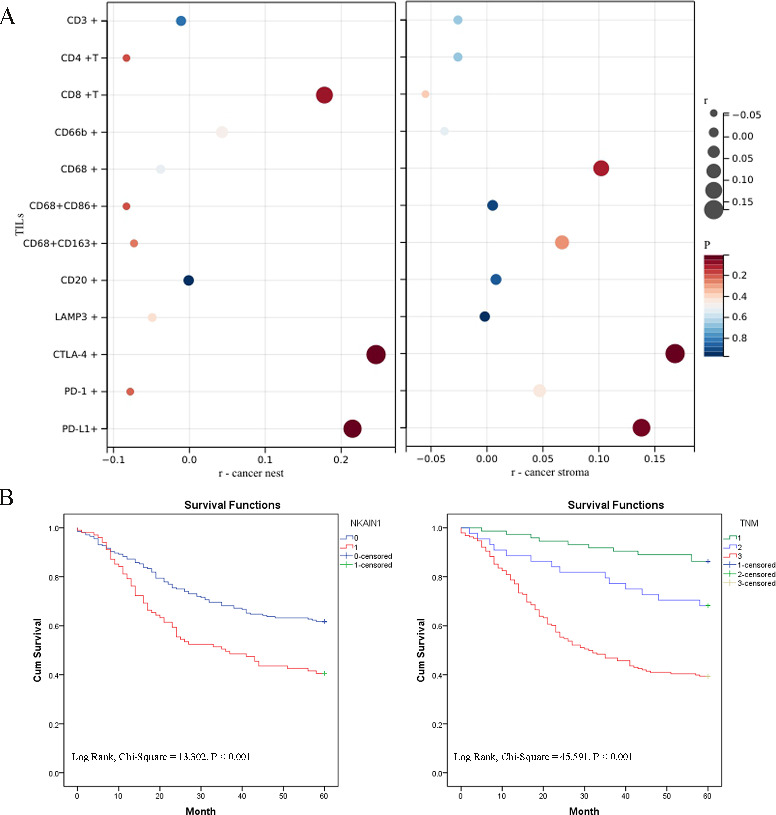
(**A**) Relationship between NKAIN1 protein expression and immune markers in gastric cancer tissues. Left, Expression of immune markers in cancer nests. Right, Expression of immune markers in stroma. (**B**) Kaplan-Meier survival analysis and log-rank test results for gastric cancer patients. Left, Survival curves comparing higher (1, red) and lower (0, blue) PLCXD2 protein expression. Right, Survival curves stratified by TNM stage: TNM 0-I (1, green line), TNM II (2, blue line), TNM III-IV (3, red line).

We conducted mIHC staining analysis on sections of human gastric cancer TMAs to assess NKAIN1 protein expression and visualize tumor-infiltrating immune cells (TIICs) and immune checkpoints (PD-1, PD-L1 and CTLA-4). Analysis of stained slides confirmed that NKAIN1 protein expression was predominantly observed in the plasma and membrane of cancer cells, showing varying degrees of positivity. NKAIN1 protein was positive staining on tumor stromal cells without statistical difference in a small number of cancer tissues. Subsequently, we investigated the correlation between NKAIN1 protein expression in cancer cells and the immune microenvironment in gastric cancerous tissue through statistical analysis. However, we did not observe a clear association between NKAIN1 protein expression and most of the TIICs (including CD3 + T cells, CD3 + CD4 + T cells, CD3 + CD8 + T cells, CD20 + B cells, CD66b + neutrophils, CD68 + macrophages, CD68 + CD86 + M1-like macrophages, CD68 + CD163 + M2-like macrophages, and Lamp3 + dendritic cells) in the TME. However, significant associations were found between NKAIN1 protein expression in cancer nests and CTLA-4 (*r* = 0.246, *P* < 0.001) as well as PD-L1 expression (*r* = 0.215, *P* < 0.001) within cancer nests, demonstrating statistical significance (see Figs. 2 and 3A and Table S1).

### Significance of NKAIN1 protein levels with patient characteristics

Using X-tile software based on the 5-year OS of stomach cancer patients, we determined a cutoff of 45 for NKAIN1 protein expression in cancer cells: scores of 0–45 were categorized as low expression, and 46–100 as high expression. In accordance with this, we observed a higher prevalence of high NKAIN1 protein expression in gastric tumoral tissues compared to non-tumoral tissue (χ^2^ = 5.712, *P* = 0.017).

To further assess the association of NKAIN1 protein expression with patient’s characteristics, we stratified the 305 gastric cancer patients into high (*n* = 101) and low (*n* = 204) NKAIN1 protein expression subgroups. We found that high NKAIN1 expression was associated with moderate HER2 expression (χ^2^ = 9.636, *P* = 0.008), deeper tumor invasion (χ^2^ = 9.438, *P* = 0.024), moderate to extensive lymph node metastases (χ^2^ = 8.632, *P* = 0.035), and advanced TNM stage (χ^2^ = 8.753, *P* = 0.013) (Table 1).

**Table 1:** Correlation of NKAIN1 expression in cancer tissues with clinicopathologic features in gastric cancer patients.

Characteristic	*n*	Lower expression (%)	High expression (%)	Pearson χ2	*P*
Total	305	204 (66.89)	101 (33.11)		
Gender				0.225	0.635
Female	93	64 (68.82)	32 (31.18)		
Male	212	140 (66.04)	72 (33.96)		
Age				0.283	0.595
<60	109	75 (68.81)	34 (31.19)		
≥ 60	196	129 (65.82)	67 (34.18)		
Differentiation				5.669	0.059
Well	11	10 (90.91)	1 (9.09)		
Middle	58	33 (56.90)	25 (43.10)		
Poor	236	161 (68.22)	75 (31.78)		
HER2				9.636	0.008*
0	212	151 (71.23)	61 (28.77)		
1+	48	23 (47.92)	25 (52.08)		
2 + 3+	22	15 (68.18)	7 (31.82)		
Unknown					
T				9.438	0.024*
Tis-T1	55	43 (78.18)	12 (21.82)		
T2	44	33 (75.00)	11 (25.00)		
T3	47	34 (72.34)	13 (27.66)		
T4	159	94 (59.12)	65 (40.88)		
N				8.632	0.035*
N0	98	75 (76.53)	23 (23.47)		
N1	46	32 (69.57)	14 (30.43)		
N2	65	36 (55.38)	29 (44.62)		
N3	96	61 (63.54)	35 (36.46)		
M				0.067	0.796
M0	298	199 (66.78)	99 (33.22)		
M1	7	5 (71.43)	2 (28.57)		
TNM stage				8.753	0.013*
0-I	73	57 (78.08)	16 (21.92)		
II	44	33 (75.00)	11 (25.00)		
III-IV	188	114 (60.64)	74 (39.36)		

*P < 0.05.

### The NKAIN1 protein expression and stomach carcinoma patient’s outcome

Using Cox regression analysis, we established a link between NKAIN1 protein expression and the survival of the patients. Univariate assessment revealed associations between OS rates and NKAIN1 protein presence (HR: 1.848, *P* < 0.001), tumor differentiation (HR: 1.838, *P* = 0.004), T (HR: 2.005, *P* < 0.001)/N (HR: 2.021, *P* < 0.001)/M (HR: 4.035, *P* = 0.001), and TNM stage (HR: 2.693, *P* < 0.001). After accounting for these factors as confounding variables in multivariate analysis, the association between NKAIN1 protein levels (HR: 1.618, *P* = 0.006) and TNM stage (HR: 2.320, *P* < 0.001) were identified as independent factors for patient survival (Table 2). Furthermore, K-M analysis with log-rank tests demonstrated that high NKAIN1 protein expression and advanced TNM stage were correlated with poorer outcomes in gastric cancer patients (Fig. 3).

**Table 2:** Univariate and multivariate analysis of prognostic factors for overall survival in gastric cancer patients.

Varible	Univariate analysis			Multivariate analysis		
	HR	*P* >|z|	95% CI	HR	*P* >|z|	95% CI
NKAIN1 expression						
High vs. low and none	1.848	<0.001*	1.319–2.590	1.618	0.006*	1.150–2.276
Age (years)						
≤ 60 vs. > 60	1.156	0.420	0.813–1.644			
Gender						
Male vs. Female	1.062	0.752	0.733–1.538			
Differentiation						
Well vs. Middle vs. Poor	1.838	0.004*	1.221–2.768	1.549	0.050	1.001–2.397
HER2						
0 vs. 1 + vs. 2 + and 3+	1.283	0.059	0.991–1.660			
T						
Tis and 1 vs. 2 vs. 3 vs. 4	2.005	<0.001*	1.635–2.459			
N						
N0 vs. N1 vs. N2 vs. N3	2.021	<0.001*	1.719–2.376			
M						
M0 vs. M1	4.035	0.001*	1.775–9.173			
TNM stage						
0 and I vs. II vs. III and IV	2.693	<0.001*	2.068–3.508	2.320	<0.001*	1.728–3.115

*P < 0.05.

## Discussion

Exploring biomarkers for stomach cancer holds significant potential for improving clinical diagnosis, treatment, prognosis, and research endeavors^22,23^. In this study, we used bioinformatics and identified NKAIN1 mRNA as a promising prognostic biomarker in human gastric carcinoma. Previous research has demonstrated the utility of NKAIN1 mRNA expression in urine sediment for early clinical diagnosis and in cancer tissue for immunotherapy in prostate cancer patients^24,25^. Moreover, NKAIN1 expression has been closely associated with OS in esophageal adenocarcinoma^26^, breast cancer^27–29^, and glioblastoma^30^.

Given that different tissue cells express varying levels of mRNA and produce proteins with distinct biological activities^15,16^, our study aimed to investigate NKAIN1 protein expression in the TME of gastric cancer. As anticipated, we observed higher NKAIN1 protein expression in gastric cancerous tissue compared to non-tumorous stomach tissues, which corroborated with the NKAIN1 mRNA levels from bioinformatics analysis.

Solid tumors, including gastric cancer, are often conceptualized as “organs” due to their complex occurrence and development^31^. This “organ” encompasses the TME, which comprises malignant tumor cells, infiltrating immune cells, and various cytokines with multifaceted functions^31^. To explore the association between NKAIN1 protein expression and the abundance of TIICs and immune checkpoints, we conducted mIHC staining using gastric cancer TMAs. Our findings revealed positive correlations between NKAIN1 protein expression and CTLA-4, as well as PD-L1, respectively.

CTLA-4, predominantly expressed in regulatory T cells, exhibits elevated levels in activated conventional T cells and serves to downregulate immune responses in cancer contexts^32,33^. PD-L1, also known as B7-H1, functions as an immunomodulatory molecule that aids tumor cells in evading anti-tumor immunity^34,35^. It serves as a checkpoint protein expressed on TIICs and cancer cells across various malignancies^36,37^, making it a potential target for clinical immunotherapy in human cancers^36,38^. The clinical utilization of monoclonal antibody immunosuppressants targeting CTLA-4 or PD-1/PD-L1 may significantly increase tumor treatment efficacy^12^. These findings underscore the potential significance of high NKAIN1 protein expression in gastric cancer tissues during clinical diagnosis and treatment, suggesting that immune checkpoint inhibitors targeting CTLA-4 or PD-1/PD-L1 could be considered to improve therapeutic outcomes.

The growth mechanism of gastric cancer is multifaceted, influenced by factors such as race, pathological type, and molecular targets^11^. Our data revealed an association between NKAIN1 protein levels, cancer invasion, and tumor stage. These findings suggest that the NKAIN1 protein may contribute to immune evasion in tumor cells, potentially in conjunction with CTLA-4 or PD-L1, promoting invasive growth and metastasis of gastric cancer. Interestingly, the NKAIN1-SERINC2 gene has been linked to alcohol dependence^39^, and alcohol consumption is significantly associated with the risk of gastric carcinoma^40^. These findings suggest a potential link between alcohol intake and NKAIN1 protein expression in cancer tissues, potentially exacerbating stomach mucosal damage. This association may occur through neurotransmitter systems or metabolic pathways^39^, ultimately facilitating the onset and progression of malignancy.

Given the variability in NKAIN1 protein expression in gastric cancer, its association with patients’ clinical characteristics and prognosis were explored. Our findings revealed that high NKAIN1 protein expression was not only significantly correlated with deeper tumor invasion and advanced TNM stage but was linked to poorer short-term prognosis. Based on the current literature reports, especially the research on the effect of NKAIN1, an oncogene in breast cancer in vitro cellular and in vivo animal experiments^29^, we speculate, based on the results of this study and the literature, that NKAIN1 may also promote the development of gastric cancer. Therefore, we propose that NKAIN1 may serve as a potential candidate biomarker for oncogenesis in gastric cancer.

There were several limitations worth mentioning. Firstly, its retrospective nature highlights the need for prospective studies, in particular, to combine the research with multi-center clinical gastric cancer samples to validate our findings. Secondly, while our results suggest a close association between NKAIN1 expression and the malignant phenotype, further investigations using molecular biological methods, knockdown or overexpression of NKAIN1, are required to elucidate the specific role of NKAIN1 in tumorigenesis. Lastly, future research could use cellular and animal models with methods such as RNA sequencing to explore the underlying mechanisms through which NKAIN1 contributes to gastric carcinoma.In conclusion, our study identifies NKAIN1 expression as a potential independent prognostic marker in gastric cancer. Positive correlations were observed between NKAIN1 protein expression and CTLA-4 and PD-L1, respectively. These findings suggest that NKAIN1 could be a promising immunotherapy target for gastric cancer.

## Supplementary Information

Below is the link to the electronic supplementary material.

Supplementary Material 1

## Data Availability

The datasets used and/or analysed during the current study available from the corresponding author on reasonable request.
