# Supplementary material for: NKAIN1 expression relates to the immune evasion and prognosis of gastric cancer
Source: Sci Rep. 2025 Dec 6;16:1012. doi: 10.1038/s41598-025-30676-0 (PMC12783303; doi:10.1038/s41598-025-30676-0)
Supplement: Supplementary file 1 — Supplementary Material 1 [file 41598_2025_30676_MOESM1_ESM.docx]

**Table S1. The correlation between NKAIN1 protein expression and immune markers in the gastric cancer tissues.**

|  | NKAIN1 protein expression | |
| --- | --- | --- |
|  | Pearson Correlation | *P* |
| CD3 + in cancer nest | -0.011 | 0.861 |
| CD3 + in cancer stroma | -0.026 | 0.668 |
| CD4+T in cancer nest | -0.083 | 0.174 |
| CD4+T in cancer stroma | -0.026 | 0.672 |
| CD8+T in cancer nest | 0.178 | 0.073 |
| in cancer stroma | -0.055 | 0.370 |
| CD66b+ in cancer nest | 0.043 | 0.479 |
| in cancer stroma | -0.038 | 0.531 |
| CD68 + in cancer nest | -0.038 | 0.530 |
| in cancer stroma | 0.102 | 0.096 |
| CD68+CD86+ in cancer nest | -0.083 | 0.175 |
| in cancer stroma | 0.005 | 0.937 |
| CD68+CD163+ in cancer nest | - 0.073 | 0.231 |
| in cancer stroma | 0.067 | 0.271 |
| CD20 in cancer nest | -0.001 | 0.987 |
| in cancer stroma | 0.008 | 0.898 |
| LAMP3 in cancer nest | -0.049 | 0.420 |
| in cancer stroma | -0.002 | 0.973 |
| CTLA4+ in cancer nest | 0.246** | < 0.001 |
| in cancer stroma | 0.168** | 0.006 |
| PD-1+ in cancer nest | -0.078 | 0.201 |
| in cancer stroma | 0.047 | 0.442 |
| PD-L1+ in cancer nest | 0.215** | < 0.001 |
| in cancer stroma | 0.138* | 0.023 |

* Correlation is significant at the 0.05 level (2-tailed).

** Correlation is significant at the 0.01 level (2-tailed).
